# A Rare Case of Isolated Left Pulmonary Agenesis in an Adult Male Patient

**DOI:** 10.7759/cureus.88201

**Published:** 2025-07-17

**Authors:** Nilanjan Sarkar, Rohit Chakravarty, Sandipan Mukhopadhyay

**Affiliations:** 1 Radiology, Tata Main Hospital, Jamshedpur, IND

**Keywords:** ct, lung, mri, pulmonary agenesis, usg

## Abstract

Pulmonary agenesis is a rare congenital condition where there is no development of pulmonary tissue beyond the carina. One or both lungs may be involved, but bilateral involvement is not compatible with extra-uterine existence. If one lung is involved, it is most commonly the left side. However, right lung involvement is more frequently associated with other inborn anomalies and severity in presentation.

Here, we report a 19-year-old male patient having complete agenesis of the left lung, presenting with recurrent episodes of nonspecific cough, without fever and dyspnea on exertion. His vitals were stable on examination, and all routine laboratory blood tests were normal. Chest auscultation revealed the absence of breath sounds in most of the left hemithorax. Chest wall movement was less compared to the right side. A chest radiograph was advised, which showed opacity in the left hemithorax, suspicious of a collapsed lung with mediastinal shift. To determine the nature of the opacity, a computed tomography (CT) scan of the chest was conducted, revealing complete agenesis of the left lung, along with ipsilateral shift of the heart and compensatory hyperinflation of the right lung.

Unilateral lung agenesis may remain asymptomatic until adulthood. Radiological investigations like chest radiograph may be useful in the diagnosis, but a CT scan of the chest can confirm the diagnosis with status of the pulmonary vasculature and bronchial tree.

## Introduction

Pulmonary agenesis is relatively rare, happening in about 1-3 in 100,000 live births [1,2]. There is a complete absence of lung tissue, bronchial tree, as well as pulmonary vessels. Bilateral involvement is not compatible with life, and with unilateral involvement, left lung agenesis is more common. Right lung agenesis is associated with worse prognosis due to the frequent association with cardiovascular malformations, tracheo-bronchial and vascular compression, due to more significant ipsilateral mediastinal displacement [3]. The patients of unilateral pulmonary agenesis usually present in infancy or early childhood with episodes of dyspnea and recurrent chest infection [4]. The cause of this malformation is not known with certainty, but investigators have proposed a relation with genetic factors like chromosomal abnormalities and vitamin A and folic acid deficiency during pregnancy [1].

In case of complete agenesis, the contralateral lung may herniate to the affected side with mediastinal shift. This may appear as pneumonic consolidation or collapse in the chest radiograph. A computed tomography (CT) scan, along with magnetic resonance imaging (MRI), can accurately diagnose this condition by confirming the absence of lung tissue [5].

We present a case of a young adult male patient having recent episodes of recurrent cough and exertional dyspnea. The diagnosis was not suspected until a chest radiograph was performed. It was confirmed by a CT scan of the chest, which showed a complete absence of the left lung.

## Case presentation

**Figure 1:**
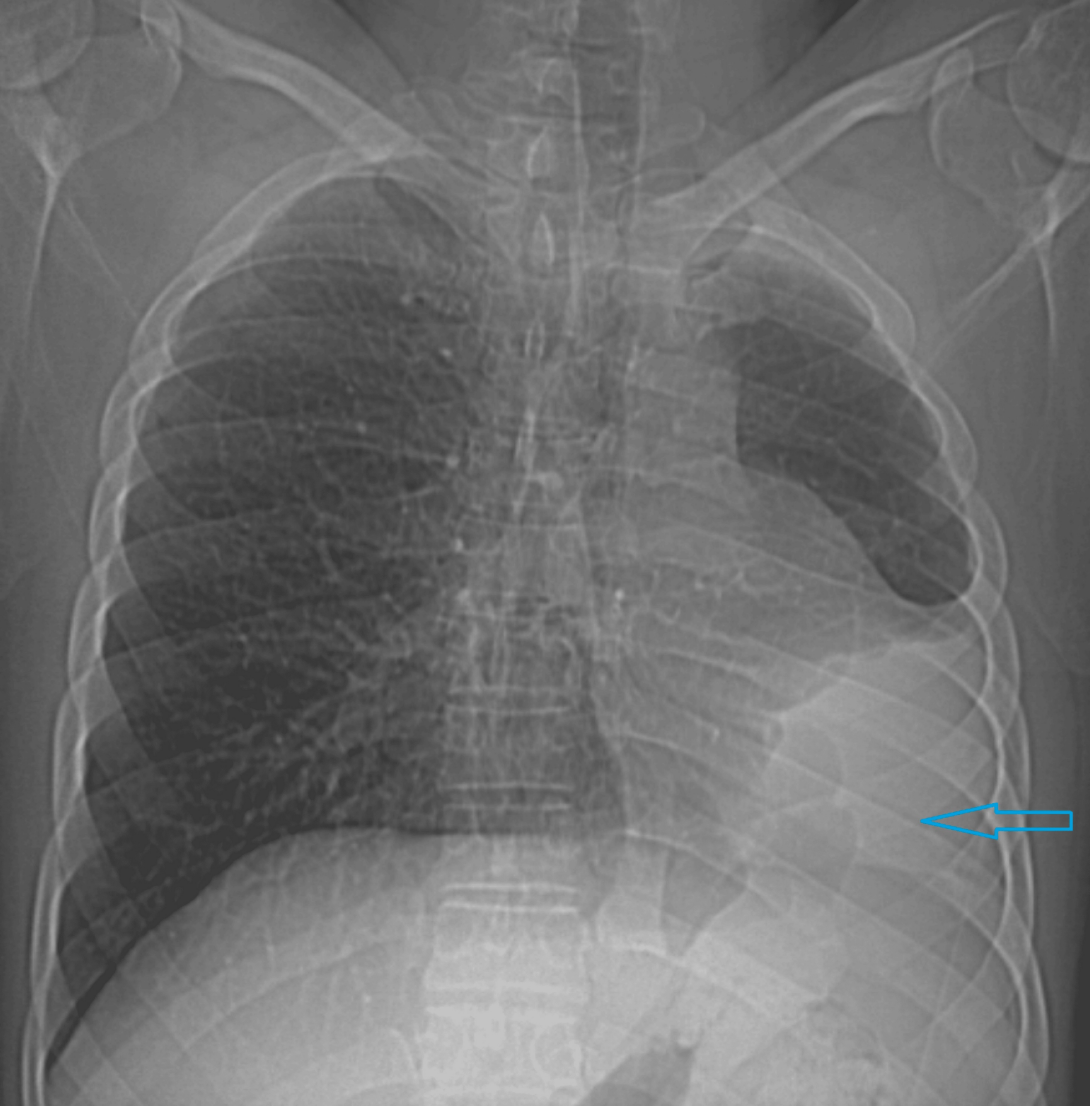
Chest radiograph in posteroanterior view shows opacity in the left lower hemithorax with ipsilateral mediastinal shift and elevated left hemidiaphragm

**Figure 2:**
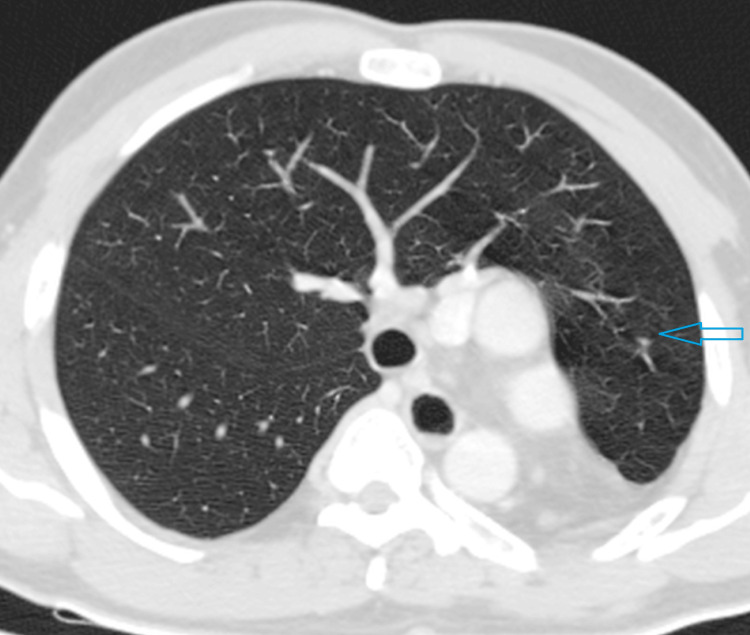
CT scan of the chest in the lung window reveals a complete absence of the left lung with compensatory hyperinflation and transmediastinal herniation of the right lung CT: computed tomography

**Figure 3:**
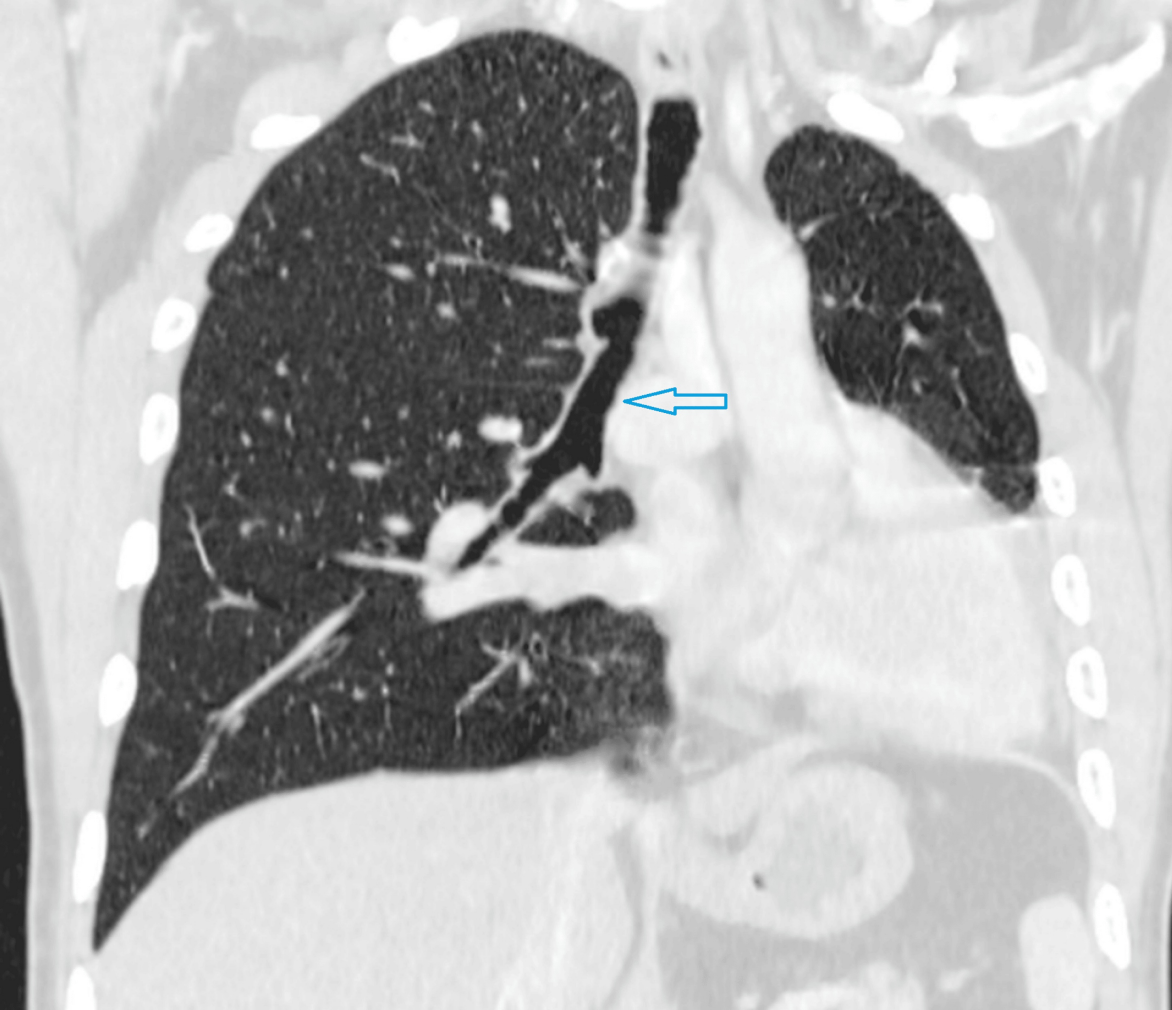
CT scan of the chest in the lung window and coronal reconstruction shows absent left main bronchus CT: computed tomography

**Figure 4:**
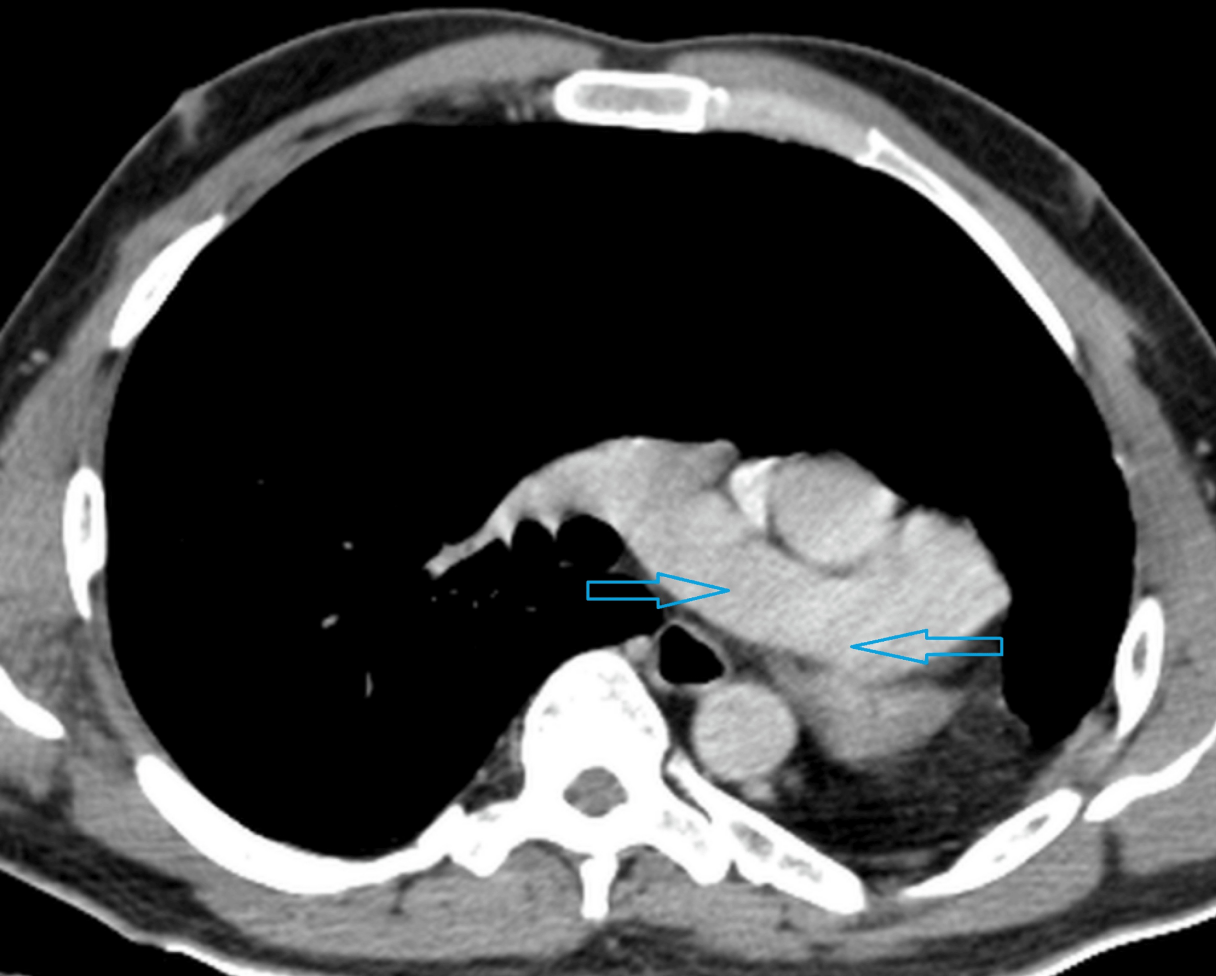
Contrast CT scan of the chest in the mediastinal window shows absence of the left pulmonary artery and normal appearing right pulmonary artery CT: computed tomography

**Figure 5:**
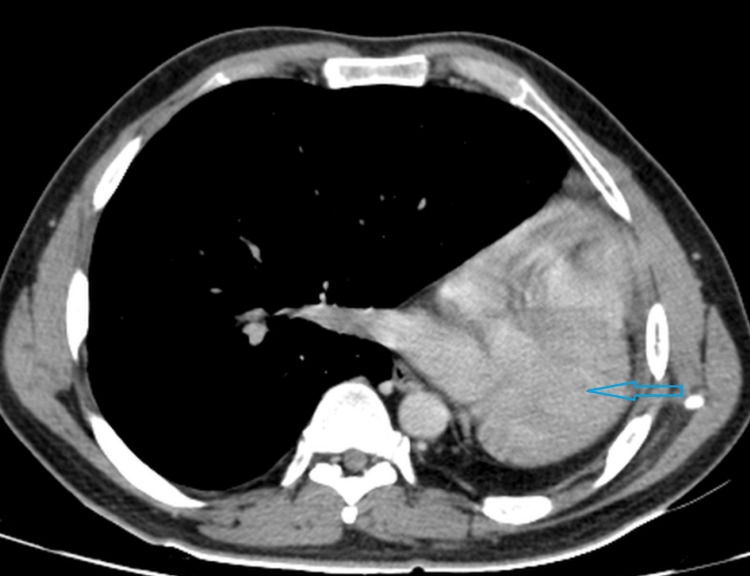
Contrast CT scan of the chest in mediastinal window shows the heart displaced in the left hemithorax along the chest wall CT: computed tomography

A 19-year-old male patient presented with recurrent episodes of cough and dyspnea triggered by minor exertion. He reported no such symptoms during childhood, with the onset occurring only in recent months. Upon general examination, he appeared to have an average build. His body temperature was normal, and his vital signs were stable. Oxygen saturation was measured at 99% in room air. Systemic examination revealed some asymmetry in the chest wall, with reduced respiratory movements on the left side. The left hemithorax was dull on percussion, and breath sounds were not audible on the left side, except in the upper zone. Normal breath sounds were noted on the right side of the chest. The apex beat was palpated in the left fifth intercostal space. Routine laboratory tests, including inflammatory markers, returned to normal. To evaluate further, a posteroanterior radiograph of the chest was advised. It revealed opacity in the left lower hemithorax, which was produced by the displaced cardiac shadow. Mediastinal structures were shifted to the left. The left hemidiaphragm was elevated, indicating lung volume loss (Figure 1). It was followed up with an intravenous contrast CT scan of the chest. There was a complete absence of the left lung with ipsilateral shift of mediastinal structures. The right lung showed compensatory hyperinflation with transmediastinal herniation of the upper and lower lobes in the left hemithorax (Figure 2). It explained the presence of breath sounds in part of the left hemithorax, although there was ipsilateral lung agenesis. The right lung was otherwise unremarkable without any evidence of active or old infection. The right main bronchus and its branches appeared normal, and the left main bronchus was completely absent (Figure 3). There was also a complete absence of the left pulmonary artery and veins (Figure 4). The heart was displaced posteriorly and laterally in the left hemithorax, right ventricle located anteriorly, and left ventricle being directly posterior to it, abutting the chest wall (Figure 5). The left hemidiaphragm was elevated, and the volume of the left hemithorax was less compared to the other side. Echocardiography correlation was found to be unremarkable. A follow-up ultrasound (USG) of the abdomen was done to rule out any associated congenital anomaly, which was also within normal limits. A diagnosis of complete agenesis of the left lung was made without any associated anomaly. Fiberoptic bronchoscopy was advised, but the patient refused. The cause for his cough was attributed to an upper respiratory tract infection without any lower tract involvement. He was provided with symptomatic medications and advised to have regular follow-ups.

## Discussion

Lung buds begin to emerge from the primitive foregut during the fourth week of embryonic life. By the fifth week, the laryngotracheal diverticulum originates from the foregut wall and subsequently divides into the right and left bronchi. As the principal bronchi encounter the developing pericardio-peritoneal canals (pleural cavity), they undergo subdivisions to form the intrapulmonary bronchial tree. This process relies on various growth factors, including retinoic acid produced by the adjacent mesoderm [6]. Any malformation during lung bud formation can lead to pulmonary agenesis or aplasia. Factors like chromosomal abnormalities, maternal intake of salicylates during pregnancy, and vitamin A deficiency may contribute to this malformation [1,2]. Lung agenesis typically presents early in life, often resulting in a fatal outcome [7]. In rare cases, patients may present later or may remain asymptomatic throughout their lives [4].

Pulmonary agenesis is broadly classified into three types [8]. Type 1, known as agenesis, is characterized by the total absence of pulmonary parenchyma and bronchus without any vascular supply. As the lung parenchyma is absent along with the bronchus, the trachea continues as the main bronchus on the opposite side, where it divides into branches. The pleural cavity is also absent on the affected side, and the contralateral lung undergoes hypertrophy due to a compensatory mechanism [3]. In type 2, also referred to as aplasia, there is a complete absence of the lung tissue, although a rudimentary bronchus is present. In type 3, known as hypoplasia, a variable amount of pulmonary tissue, bronchus, and vessels are present. Left-sided involvement is more common, but right lung agenesis is associated with poorer outcome due to frequent association with cardiovascular and genitourinary anomalies [9]. Among the cardiovascular anomalies, atrial septal defect is most frequently encountered [10]. Our case falls under type 1 (agenesis), without any other associated congenital anomaly.

Antenatal USG can diagnose lung agenesis or hypoplasia by measuring lung volume. MRI is superior in this regard due to its large field of view and high soft tissue contrast, compared to USG. Maternal body habitus, fetal position, and amount of amniotic fluid do not significantly affect optimum visualization in MRI [11].

The cornerstone of postnatal diagnosis typically involves cross-sectional imaging studies, such as a CT scan. These scans can confirm the absence or variable presence of lung parenchyma, bronchial tree, and pulmonary vasculature. Additionally, any related abdominal abnormality can also be assessed at the same time. CT or MR angiography is considered the ideal imaging modality, particularly for evaluating the status of the pulmonary vasculature. Detailed knowledge of the vascular anatomy is also important for those patients who may require embolization [12]. CT scan not only confirms the diagnosis but also identifies any active pulmonary infections or long-term sequelae, such as fibrosis or bronchiectasis [7,9]. Bronchial tree anatomy, especially the status of the bronchus on the affected side, can be evaluated by bronchoscopy [7]. Patients who are asymptomatic do not require any active intervention if there are no associated anomalies. However, surgical treatment such as diaphragmatic translocation and aortopexy may be necessary in cases where tracheal compression leads to respiratory failure due to the rotation of the heart or aortic arch [4,11]. Recurrent respiratory distress may arise from the retention of bronchial secretions accompanied by superadded infection, which can often be managed conservatively [9]. In the absence of associated anomalies, the long-term prognosis for these patients is generally favorable [13].

## Conclusions

Pulmonary agenesis is a congenital condition that may present in early childhood but can remain undetected until adulthood. Some patients may even remain asymptomatic throughout their lives. Clinicians and radiologists should consider the diagnosis in patients having unilateral opacity in the hemithorax. Diagnosis can occur in utero through antenatal USG or MRI scans and later in life through imaging techniques such as CT scans. Associated anomalies often influence the outcomes for patients. Surgical intervention is generally not necessary unless there is significant airway or vascular compression. Treatment is based on the patient's symptoms, with recommendations for a regular follow-up. Increased awareness of this condition is essential, as an initial chest radiograph may resemble lung collapse or effusion. Sectional imaging remains the cornerstone of diagnosing this condition.
